# Unraveling the molecular landscape of breast muscle development in domestic Yuzhong pigeons and European meat pigeon: Insights from Iso-seq and RNA-seq analysis

**DOI:** 10.1371/journal.pone.0305907

**Published:** 2024-07-25

**Authors:** Pengkun Yang, Xinghui Song, Liheng Zhang, Xinlei Wang, Zhanbing Han, Runzhi Wang, Mingjun Yang, Peiyao Liu, Zhen Zhang

**Affiliations:** 1 College of Animal Science and Technology, Henan University of Animal Husbandry and Economy, Zhengzhou, China; 2 Nanjing Institute of Animal Husbandry and Poultry Science, Nanjing, China; 3 Henan Tiancheng Pigeon Industry Co., Ltd, Pingdingshan, China; University of Hawai’i at Manoa, UNITED STATES

## Abstract

The mechanisms governing gene regulation in domestic Yuzhong pigeon breast muscle development remain largely elusive. Here, we conducted a comparative analysis using Iso-seq and RNA-seq data from domestic Yuzhong pigeons and European meat pigeons to uncover signaling pathways and genes involved in breast muscle development. The Iso-seq data from domestic Yuzhong pigeons yielded 131,377,075 subreads, resulting in 16,587 non-redundant high-quality full-length transcripts post-correction. Furthermore, utilizing pfam, CPC, PLEK, and CPAT, we predicted 5575, 4973, 2333, and 4336 lncRNAs, respectively. Notably, several genes potentially implicated in breast muscle development were identified, including tropomyosin beta chain, myosin regulatory light chain 2, and myosin binding protein C. KEGG enrichment analysis revealed critical signaling pathways in breast muscle development, spanning carbon metabolism, biosynthesis of amino acids, glycolysis/gluconeogenesis, estrogen signaling, PI3K-AKT signaling, protein processing in the endoplasmic reticulum, oxidative phosphorylation, pentose phosphate pathway, fructose and mannose metabolism, and tight junctions. These findings offer insights into the biological processes driving breast muscle development in domestic Yuzhong pigeon, contributing to our understanding of this complex phenomenon.

## Introduction

Meat remains a cornerstone of human diets, offering a cost-effective reservoir of proteins, long-chain fatty acids, essential vitamins, minerals, and trace elements [1]. Pigeon meat distinguishes itself with its rich protein content, ample essential amino acids and minerals, low cholesterol levels, and a significant presence of unsaturated fatty acids, rendering it an optimal and healthful dietary option [2, 3]. In China, pigeon meat enjoys the reputation of being ‘animal ginseng’, esteemed for its superior nutritional composition, attracting considerable interest among consumers [4].

Measuring meat production efficiency is essential for evaluating the economic viability of pigeon breeds, with the growth of breast muscle being particularly influential in overall meat yield [5]. Meeting consumer demands necessitates optimizing meat production. However, research on genetic regulation in pigeons lags behind that of other poultry species [6]. Therefore, investigating the molecular mechanisms governing breast muscle development in pigeons is imperative. Such insights are essential for harnessing molecular breeding techniques to improve breast meat production efficiency.

Meat quality encompasses a range of factors such as tenderness, intramuscular fat content, muscle fiber composition, and meat color, many of which have modest heritability levels [7, 8]. To address this, various molecular biology techniques have emerged to enhance meat quality through molecular breeding methods. Current research focuses on unraveling the molecular mechanisms underlying meat quality, providing essential insights for molecular selection of economically important traits [9].

Transcriptome sequencing and analysis are invaluable tools in biological research, offering insights into the intricate molecular mechanisms governing various biological processes [10]. These techniques reveal transcript levels linked to essential physiological and biochemical functions, including tissue growth, immunity, sex determination, and development [11, 12]. While traditional RNA-seq methods face challenges with short read lengths, particularly in species without reference genomes, the advent of Sin-gle-Molecular Real-Time (SMRT) Isoform Sequencing (Iso-seq) addresses this limitation by generating kilobase-long sequencing reads [13]. Iso-seq technology revolutionizes transcriptome analysis, allowing researchers to explore the transcriptomes of diverse livestock and poultry species in unprecedented detail, thereby advancing our understanding of molecular biology [14, 15]. This progress holds significant promise for enhancing livestock and poultry breeding programs.

Numerous studies have utilized a combination of Iso-seq and RNA-seq techniques to elucidate regulatory mechanisms and identify pivotal genes and signaling pathways involved in muscle development. For instance, a study applied these methods to investigate alternative splicing in porcine skeletal muscle, resulting in updated genome annotations [9]. Similarly, hybrid sequencing (Iso-seq and RNA-seq) was employed to unveil skeletal muscle genes governing development and meat quality in goats, enriching our knowledge of transcriptomic diversity linked to meat quality and muscle development [16]. Another study focused on lamb meat tenderness, utilizing Iso-seq and RNA-seq to explore novel isoforms and their regulatory pathways, leading to the identification of isoforms associated with meat tenderness and uncovering potential regulatory mechanisms [17].

The Yuzhong pigeon, also referred to as Wuyang pigeon, stands as a native breed in Henan province, China, holding significant importance as a local meat source [18]. Recent research has highlighted the remarkable qualities of its breast muscle, including favorable flavor, tenderness, ruddy color, and resistance to rancidity [18]. Moreover, the Yuzhong pigeon boasts a commendable nutritional profile, featuring a balanced amino acid composition and elevated fatty acid levels, rendering it an ideal option for low-fat, high-protein meat [18]. Despite its local significance, the breed is currently in the stage of resource collection and preservation, with limited documentation on its breed characteristics and meat quality. Thus, there’s a pressing need to delve into the superior meat quality traits of Yuzhong pigeons, laying the groundwork for conservation efforts and the development of new high-quality meat pigeon varieties.

**Fig 1:**
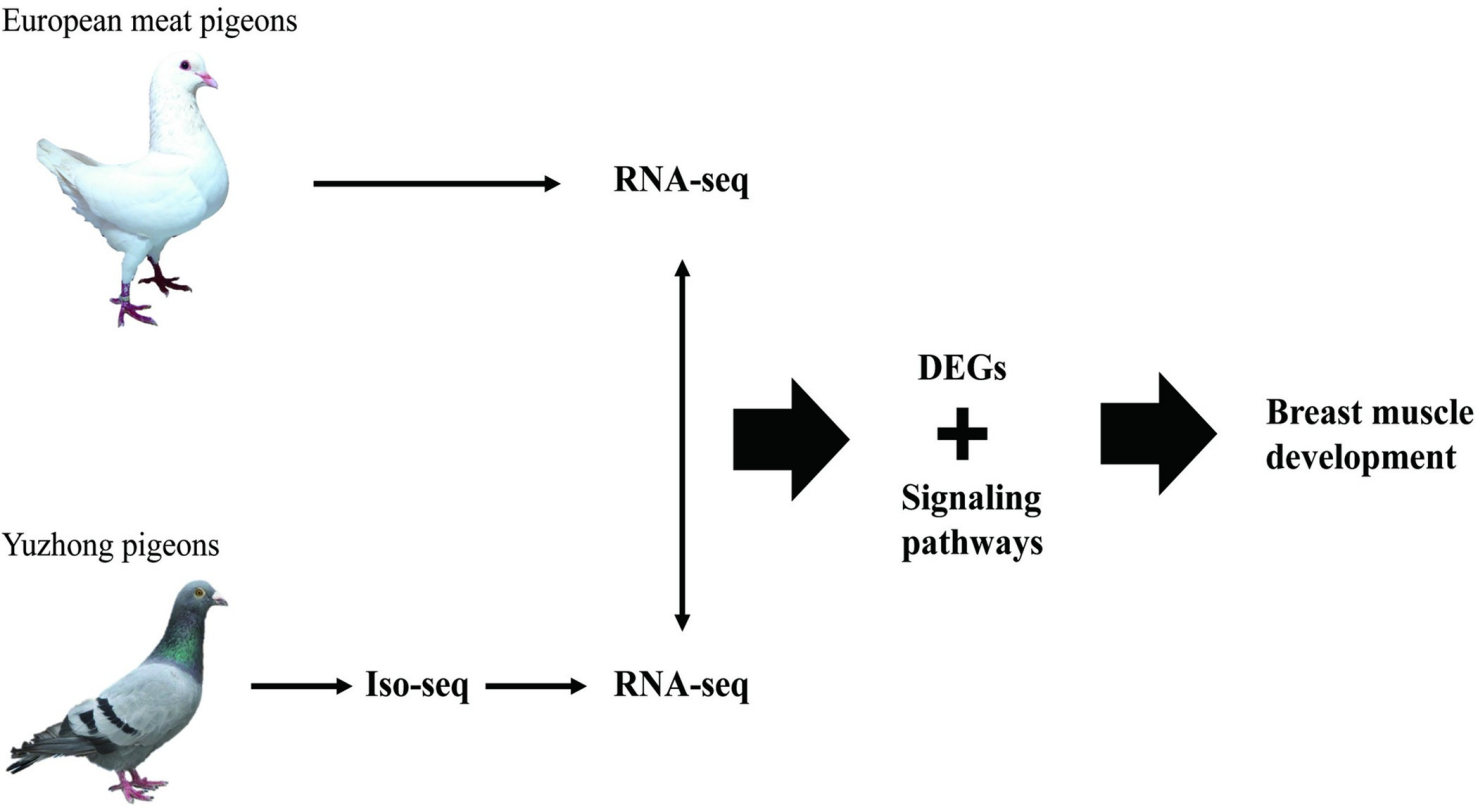
The research flowchart of the Iso-seq and RNA-seq analyses of breast muscle development.

The breast muscle constitutes the primary edible portion of meat pigeons, necessitating a thorough understanding of the genetic mechanisms underlying the development of desirable traits in these birds. To address this, we employed a comprehensive approach integrating Iso-seq and RNA-seq profiling techniques. Our objective was to elucidate the genetic determinants influencing breast muscle development in both European meat pigeons and domestic Yuzhong pigeons, aiming to pinpoint key candidate genes associated with favorable traits. By amalgamating Iso-seq and RNA-seq data, we gained a comprehensive view, significantly augmenting our comprehension of the genetic architecture linked to breast muscle development. Our investigation focused on two distinct pigeon populations: European meat pigeons and domestic Yuzhong pigeons. Through a comparative analysis, we aimed to uncover shared and divergent genetic elements influencing this specific trait across these groups. As we unveiled the intricate genetic network governing breast muscle development in pigeons, our study contributed significantly to the broader understanding of avian biology. The insights garnered from this research offer novel avenues for the breeding of meat pigeon varieties, providing a solid framework for molecular breeding and the utilization of local meat pigeon resources. The research flowchart is depicted in Fig 1.

## Material and methods

### Ethical statement

All experiments in this study were carried out following a protocol approved by the Institutional Animal Care and Use Committee (IACUC) of Henan University of Animal Husbandry and Economy (HENAU) in China. The ethical approval code is HNUAHEER 23104.

### Experimental animals and breast muscle collection

Six European meat pigeons (S1 Fig), weighing between 390 and 560 g, and six Yuzhong pigeons (S2 Fig), weighing between 360 and 430 g, were obtained from Henan Tiancheng Pigeon Industry Co. Ltd, located in Henan province. Both pigeon species were anesthetized prior to slaughter using intravenous (IV) injection of pentobarbital sodium, followed by exsanguination, without scalding. Samples were collected by removing the breast muscles from both sides. The collected breast muscle samples were promptly submerged in liquid nitrogen (-196°C) for 24 hours for preservation and subsequently stored at -80°C until total RNA isolation.

### Total RNA isolation and RNA-seq

Total RNA isolation for each sample was performed using TRIzol Reagent (Takara, Dalian, China), according to the manufacturer’s protocol. Following isolation, the RNA samples underwent treatment with gDNA eraser (Takara, Dalian, China) to remove any genomic DNA contamination. The concentration, quality, and purity of the isolated RNA were evaluated using a Nanodrop ND-2000 spectrophotometer (Thermo Fisher Scientific, MA, USA), Agilent Bioanalyzer 2100 (Agilent Technologies, CA, USA), and Qubit 2.0 fluorometer (Life Technologies, CA, USA). The RNA extracted from breast muscle samples of both European meat pigeons and Yuzhong pigeons underwent stringent quality assessments, ensuring an OD260/280 ratio between 2.0 and 2.2 and a minimum RNA Integrity Number (RIN) of 8.0, to guarantee high-quality samples for RNA-seq analysis. Subsequently, the RNA samples were reverse-transcribed into complementary DNA (cDNA) and sequenced using an Illumina HiSeqTM 2300 sequencer (Illumina, San Diego, USA) by Frasergen Biotechnology (Wuhan, China), resulting in the construction of a total of 12 RNA-seq libraries.

### De novo assembly and annotation

Following RNA-seq, the generated raw reads underwent quality assessment and visualization using FastQC (version 0.11.05) to ensure data integrity (http://www.bioinformatics.babraham.ac.uk/projects/fastqc). Reads with low quality and those containing adapters were eliminated using the NGS QC toolkit [19]. De novo assembly was performed using the Trinity platform with kmer = 25 [20]. The TGI Clustering Tool (version 2.1) was employed to remove instances of sequence splicing and redundancy, resulting in the acquisition of non-redundant unigenes [21]. Subsequently, unigene annotation was carried out using BLASTx (version 2.2.26) with an E-value threshold of 10–5 for the comparison and classification of the identified unigenes. The quantification of reads aligning to genes was performed using the Bowtie2 software [22]. Analysis of Differentially Expressed Genes (DEGs) utilized the R package DeSeq2 [23]. Gene expression levels were predicted based on fragments per kilobase transcript sequence per million base pairs sequenced (FPKM), calculated through base mean values. Statistical significance was determined by q-value, corresponding to the false discovery rate (FDR), with thresholds of q-value < 0.05 and | log2 (fold change) | > 1 indicating significant differential ex-pression. To identify functional enrichment of the identified DEGs, a Gene Ontology (GO) enrichment analysis was conducted using the GOseq package. Significantly enriched pathways were identified using KOBAS 2.0, maintaining an FDR threshold of < 0.05 [24].

### Iso-seq cDNA library construction and PacBio SMRT sequencing

The breast muscle, leg muscle, liver, abdominal fat, skin fat, hypothalamus, pituitary, and cecum tissues from Yuzhong pigeons were harvested, and RNA extraction was performed to create a single Iso-seq cDNA library. An equal amount of RNA was combined from each sample for library construction. Subsequently, 3 μg of total RNA underwent reverse transcription using the Clontech SMARTer PCR cDNA synthesis kit (Takara, Dalian, China) to generate double-stranded cDNA. Size selection of the PCR products was carried out using the BluePippin system. The size selection was performed using the Size Selection system (Sage Science, MA, USA), targeting fragments within the 0.5–0.6 kb range. For each SMRTbell library preparation, 1 μg of size-selected cDNA was me-ticulously utilized with the Pacific BioSciences SMRTbell template prep kit (PacBio, CA, USA). The pivotal Iso-sequencing process was carried out using the state-of-the-art Pacific Biosciences Sequel II platform (Frasergen, Wuhan, China).

### Error correction of obtained Iso-seq reads

SMRTlink 10.0 software was employed for processing the raw polymerase reads, involving the elimination of low-quality reads and adapters to obtain refined post-filter polymerase reads. The Reads of Insert (ROIs) obtained were categorized into full-length (FL) and non-full-length (nFL) transcript sequences. A three-step error correction strategy was implemented to enhance the accuracy of full-length transcripts. By utilizing a circular sequencing approach with multiple passes, Circular Consensus Sequences (CCSs) underwent self-correction. Following this, Full-Length Non-Chimeric (FLNC) reads underwent non-redundancy treatment and clustering using the ICE Quiver algorithm, with subsequent Arrow polishing to generate high-quality full-length consensus sequences. These polished full-length consensus sequences underwent further correction and reduction of redundancy through alignment with Illumina short reads using the LoRDEC tool [25]. Additional refinement was accomplished using the CD-Hit program, configured with a -c 0.95 parameter [26]. This meticulous process yielded highly precise and non-redundant full-length unigenes at the isoform level, forming the foundational basis for subsequent analyses, ensuring the integrity and reliability of the study’s outcomes.

### Iso-seq functional annotation

The non-redundant transcripts underwent comprehensive annotation across various databases, including NCBI non-redundant protein sequences (NR), NCBI non-redundant nucleotide sequences (NT), Gene Ontology (GO), Kyoto Encyclopedia of Genes and Genomes (KEGG), Cluster of Orthologous Groups of proteins (COG), Swiss-Prot, and Protein family (Pfam). The BLAST software was employed with a stringent criterion of an E value < 1 × 10–10 to ensure accuracy in NT database annotation. Additionally, the Diamond BLASTX strategy, with an E value < 1 × 10–10 parameter, was executed to enhance the robustness of annotations [27]. For Pfam database annotation, the Hmmscan algorithm was utilized. Simultaneously, WEGO was applied to categorize GO functions, providing a systematic classification of gene functions within a broader biological context [28]. This comprehensive annotation approach ensures a thorough understanding of the functional landscape of the non-redundant transcripts.

### Long non-coding RNA, SSR analysis, and CDS prediction

The Coding-Non-Coding Index (CNCI), the Coding Potential Calculator (CPC), and the Coding Potential Assessment Tool (CPAT) were utilized to identify potential Long Non-Coding RNAs (LncRNAs) from transcripts exceeding 200 nucleotides in length and featuring more than two exons. The TransDecoder (V3.0.1) software was then employed to identify potential Coding Sequence (CDS) regions within transcripts, including Open Reading Frame (ORF) length, amino acid sequence, and protein domain sequences within the Pfam database. Validation of CDS was further conducted using the ANGLE software with both fault-tolerant and error-free modes [29]. Additionally, the MISA software was utilized to identify Simple Sequence Repeats (SSRs) within the analyzed transcripts. This comprehensive approach ensures the accurate identification and validation of potential LncRNAs and associated features.

### Real-time quantitative PCR assay

The genes *MYOT*, *SELENOP*, *FKBP5*, *ALDOA*, *Ube2b*, *MYH*, *MYL2*, *and GAPDH* were specifically targeted and amplified using primers detailed in Table 1, with 18S chosen as the reference gene. The first cDNA strand was synthesized through reverse transcription employing the Prime Script RT Reagent kit with a genomic DNA (gDNA) eraser from TaKaRa Bio Inc., Dalian, China. Gene expression levels were quantified using the Quantstudio 6 Flex from Applied Biosystems, Thermo Fisher, USA. For qRT-PCR analysis, a 96-well plate was utilized, with each well containing a 20 μL reaction mixture comprising 10 μL SYBR Premix Ex Taq II from TaKaRa Bio Inc., Dalian, China, 0.4 μL of each forward and reverse primer (10 μM), 2 μL of cDNA template, and 7.2 μL of DEPC water. The qRT-PCR protocol involved an initial denaturation step at 95°C for 5 min, followed by 40 cycles of amplification at 95°C for 10 s and 72°C for 15 s. Gene expression levels were determined in triplicate and analyzed using the 2^-△△Ct^ method.

**Table 1:** Primers used for expression level quantification by qRT-PCR.

Gene name	Primer sequence (5’ to 3’)
*MYOT*-F	TACAGCCAGACTTGAATG
*MYOT*-R	AATCCTTCCAGTATTGTCAT
*SELENOP*-F	GGGGAGCAAAGGATAAAG
*SELENOP*-R	CTTGCAGTTCTGTGACTC
*FKBP5*-F	GGAAGGAGAAGAGAATGTC
*FKBP5*-R	GATCACAATTCAGGTCAGA
*ALDOA*-F	GAGAACACGGAGGAGAAC
*ALDOA*-R	CTTCTGGTAGAGCGTCTC
*Ube2b*-F	AATAAGCCTCCAACTGTTAG
*Ube2b*-R	ATTAGGTTCATCAAGTAGAGAC
*MYH*-F	ATACGAGACTGACGCTAT
*MYH*-R	GCTCCACATCAATCATCA
*MYL2*-F	GCTTGAAGAGGAGTTGAA
*MYL2*-R	CGGTCAGGACTTTAATCTC
*GAPDH*-F	ATGCTGGTGCTGAATATG
*GAPDH*-R	CAGAGATGATAACACGCTTA
18S-F	CGGTCGGCGTCCAACTTCTTAG
18S-R	TAGGCACAAGCTGACCAGTCA

### Statistical analysis

Statistical analysis was performed using SPSS 19.0 software from IBM Analytics, VA, USA. Levene’s test was employed to assess the homogeneity of variances, while the normal distribution of data residuals was verified using the Shapiro-Wilk test. To evaluate significant differences between samples, one-way analysis of variance (ANOVA) and Tukey-HSD tests were applied, with a statistical significance threshold set at P < 0.05.

## Results

### Iso-seq and RNA-seq analysis

**Fig 2:**
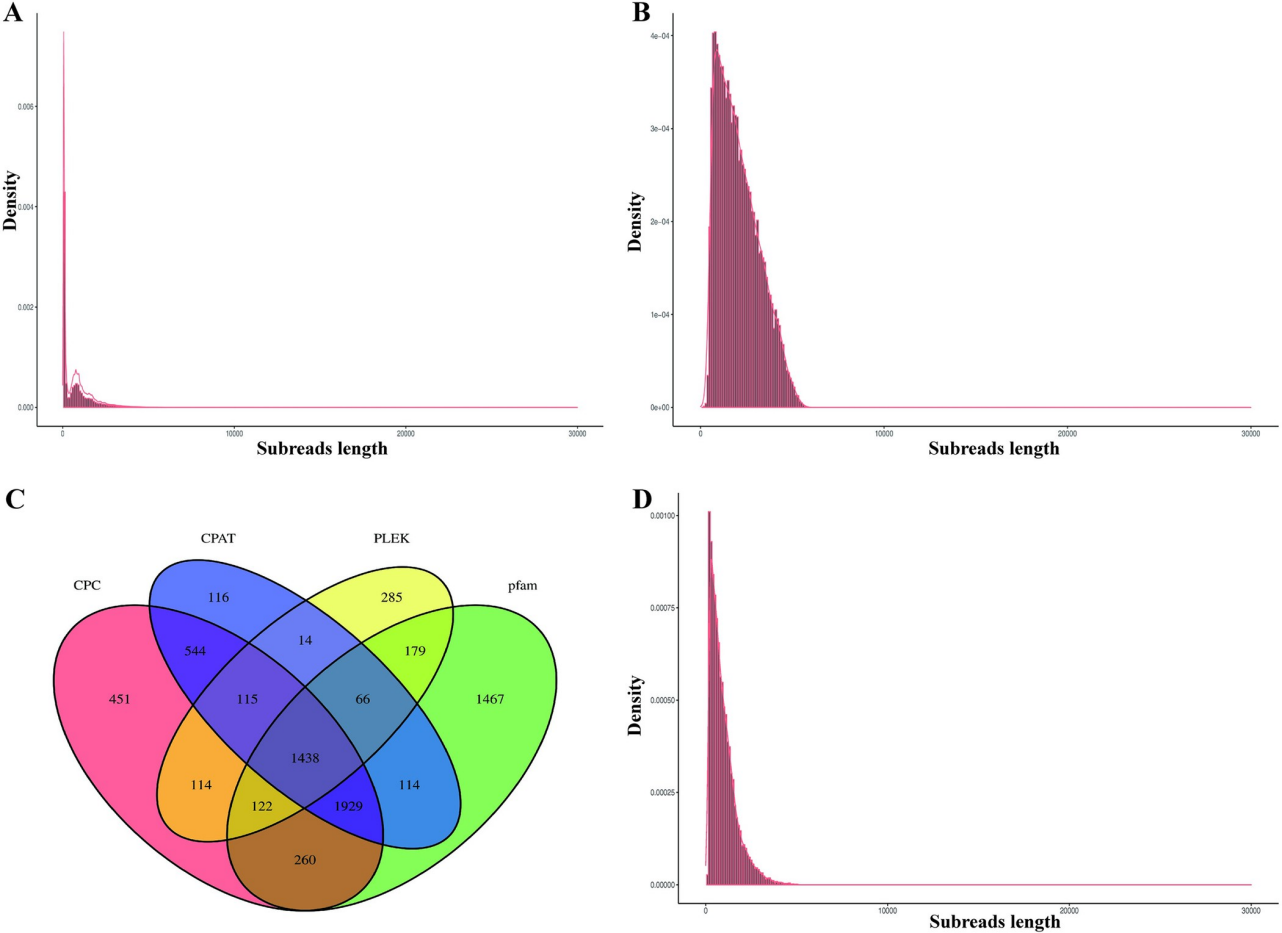
Statistical analysis of Iso-seq in domestic Yuzhong pigeon. (A) The distribution of Iso-seq subreads length. (B) The non-redundant high-quality full-length transcripts generated. (C) The four-way Venn diagram of lncRNAs prediction. (D) The length distribution of identified ORF.

The Pacific Biosciences Sequel platform was employed to generate a comprehensive full-length transcriptome of Yuzhong pigeons. RNA samples were carefully extracted from various tissues, including breast muscle, leg muscle, liver, abdominal fat, skin fat, hypothalamus, pituitary, and cecum. Subsequently, these RNA samples were pooled and sequenced using PacBio Iso-seq technology, yielding 131,377,075 subreads, with an average length of approximately 743 base pairs (bp). The distribution of subreads length is depicted in Fig 2A. Subsequent analysis identified 689,381 Circular Consensus Sequence (CCS) reads and 524,794 Full-Length Non-Chimeric (FLNC) reads. Through self- and NGS-corrected procedures, this led to 33,451 self-corrected FLNC and 39,870 NGS-corrected FLNC reads. A CD-Hit analysis of these reads generated 16,587 non-redundant high-quality full-length transcripts (Fig 2B). Table 2 provides a comprehensive overview of the Iso-seq statistics for the domestic Yuzhong pigeon, while S3–S6 Figs. illustrate the distribution of CCS reads length, FLNC reads length, NGS-corrected FLNC reads length, and self-corrected FLNC reads length, respectively. The raw sequencing data has been uploaded, and its accession number is PRJNA1103993.

**Table 2:** Comprehensive overview of the Iso-seq statistics for the European meat pigeon and domestic Yuzhong pigeon.

Parameters	Subreads	CCS reads	FLNC	Self-corrected FLNC	NGS-corrected FLNC	Non-redundant full-length transcript
Seq. number	131,377,075	689,381	524,794	33,451	39,870	16,587
Total length (bp)	97,718,004,619	1,110,672,219	863,659,780	61,536,726	60,011,899	34,563,999
< 1 kb	96,902,115	238,369	161,034	8,279	14,030	3,260
>1 kb & < 2 kb	23,494,602	249,566	213,259	12,653	16,982	5,623
>2 kb & < 3 kb	7,138,164	120,916	96,917	7,518	5,780	4,048
> 3 kb	3,842,194	80,530	53,584	5,001	3,078	3,656
N50	1,563	2,172	2,014	2,308	1,760	2,651
N90	576	927	875	1,006	813	1,156
Mean	743	1,611	1,645	1,839	1,505	2,083
Median	376	1,398	1,443	1,624	1,274	1,889
Max	268,870	24,889	6,306	5,647	5,795	5,795
Min	51	51	71	106	27	182

To enhance the characterization of gene expression, Illumina RNA sequencing was conducted on 12 breast muscle libraries derived from Yuzhong pigeons (6 individuals) and European meat pigeons (6 individuals). This RNA-seq generated 44–81 million reads, with Q20 and Q30 content ranging from 96–97% and 90–92%, respectively. Additionally, the GC content for the RNA-seq ranged from 49–51%, while the total alignment ratio ranged from 72.26–76.30%. Table 3 presents the summarized statistics for the RNA-seq of both European meat pigeon and domestic Yuzhong pigeon.

**Table 3:** Summarized statistics for the RNA-seq of both European meat pigeon and domestic Yuzhong pigeon.

Sample	Total reads	Q20 (%)	Q30 (%)	GC content (%)	Total alignment ratio (%)
O1	52,132,197	97	91	51	75.05
O2	54,897,579	96	90	50	75.33
O3	67,582,847	97	92	51	73.01
O4	66,667,966	97	91	51	72.63
O5	57,340,471	97	91	50	73.89
O6	76,781,926	97	91	50	72.89
W1	57,882,456	97	90	50	76.12
W2	60,397,236	97	91	49	72.26
W3	81,727,337	97	92	51	74.80
W4	81,365,211	97	92	50	72.75
W5	44,163,676	97	91	50	73.99
W6	51,842,289	97	91	50	76.30

### LncRNA, ORF prediction, and functional annotation

To identify and annotate transcripts with potential functional roles as long non-coding RNAs (lncRNAs) in various biological processes, prediction analysis was conducted on domestic Yuzhong pigeon using tools such as pfam, CPC, PLEK, and CPAT, resulting in predictions of 5575, 4973, 2333, and 4336 lncRNAs, respectively. To showcase the overlap in predictions, a four-way Venn diagram (Fig 2C) visually represents the intersection of lncRNA predictions from pfam, CPC, PLEK, and CPAT analyses. Subsequently, the analysis of Open Reading Frames (ORFs) was conducted using TransDecoder, identifying a total of 15,975 ORFs with a cumulative length of 15,145,779 bp. Notably, the majority of identified ORFs, totaling 10,097, exhibited a length below 1 kb. The N50 value of 1299 bp and a mean length of 948 bp provide additional insights into the distribution characteristics of these identified ORFs. Fig 2D elucidates the length distribution of the identified ORFs, contributing to a comprehensive understanding of their structural features.

**Fig 3:**
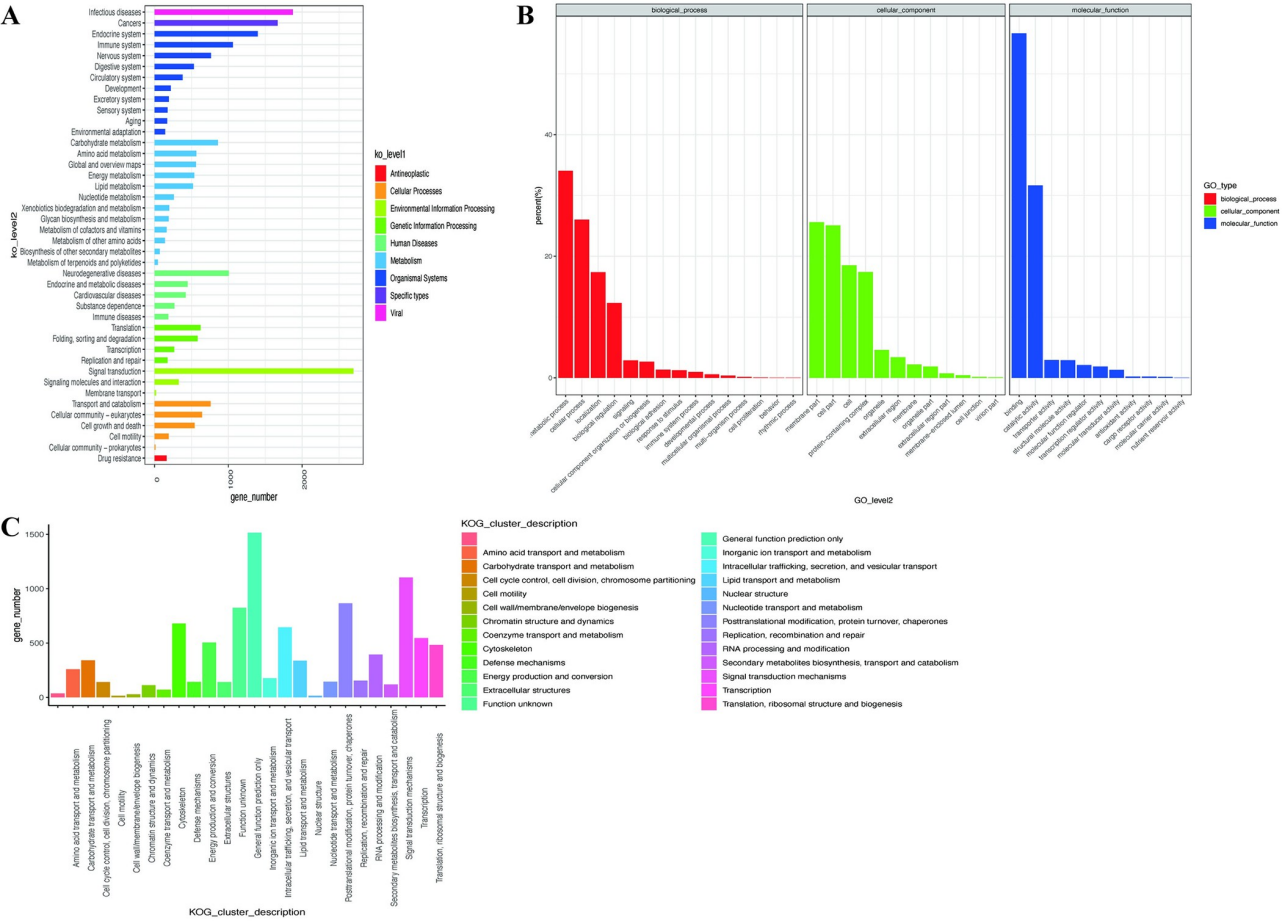
Functional annotation and classification of identified Iso-seq transcript in domestic Yuzhong pigeon. (A) Kyoto Encyclopedia of Genes and Genomes (KEGG). (B) Gene Ontology (GO). (C) Eukaryotic Orthology Groups (KOG).

To assign biological functions to the identified transcripts, they underwent annotation across seven databases: Nr, pfam, GO, KEGG, KOG, SwissProt, and TrEMBL. The annotations yielded specific counts for each database, with the numbers of annotated transcripts for the domestic Yuzhong pigeon being 13,155, 11,246, 7,064, 9,300, 9,789, 12,431, and 13,092, respectively. To provide a visual representation of the functional annotation outcomes, Fig 3A–3C present the KEGG, GO, and KOG annotations of the identified transcripts in the domestic Yuzhong pigeon.

### Differential expression gene (DEGs) analysis

**Fig 4:**
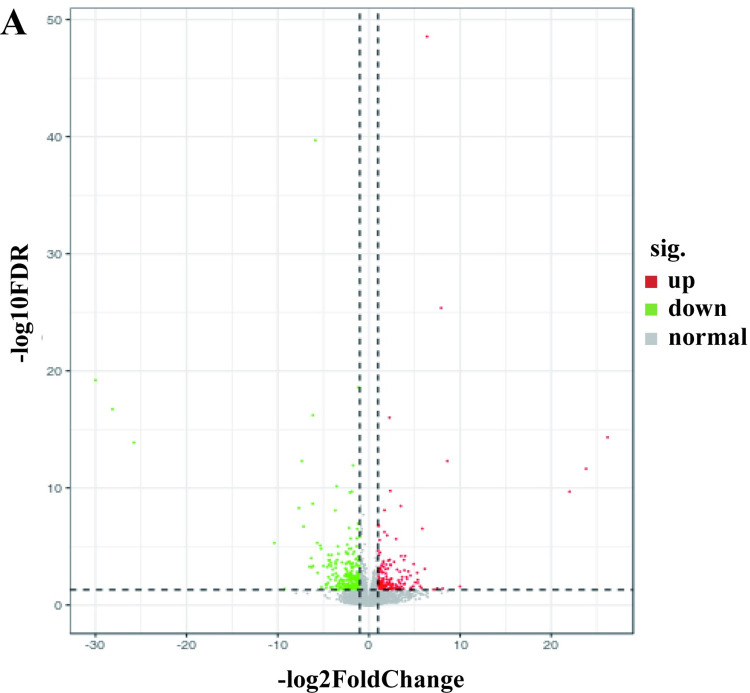
The up- and down-regulated DEGs identified in the comparison between European meat pigeons and domestic Yuzhong pigeons. The expression volcano plot of the up- and down-regulated of identified DEGs. Indication, sig.: signal; up: up-regulated; down: down-regulated.

DEGs analysis was performed to identify genes showing statistically significant differences in expression levels between Yuzhong pigeon and European meat pigeon. The gene expression analysis revealed a total of 443 differentially expressed genes (DEGs) between European meat pigeons and domestic Yuzhong pigeons (S1 Table). Among these, 178 genes were significantly up-regulated, while 265 genes showed down-regulation. Additionally, volcano plots were constructed to visually depict the expression trends, effectively illustrating the extent of up- and down-regulated DEGs (Fig 4). These results contribute to an enhanced understanding of changes in gene expression and their significance during breast meat development.

### The signaling pathway involved in breast muscle development

**Fig 5:**
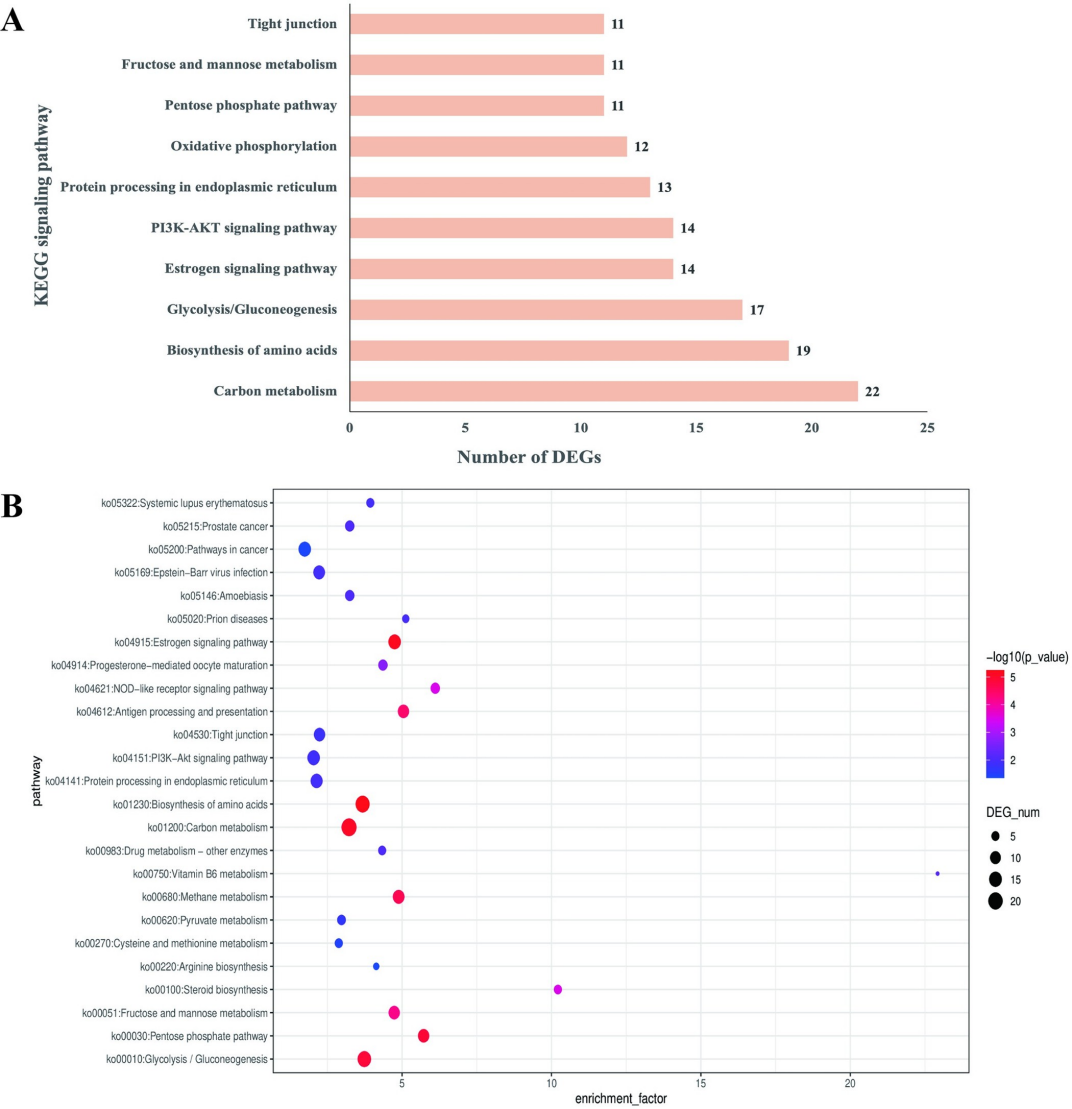
The KEGG signaling pathway and the number of DEGs involved in the breast muscle development. (A) Bar plot of signaling pathways and the number of implicated genes. (B) Bubble plot of signaling pathway and enrichment factor. Indication, DEG_num: differential expression gene (DEG) number.

To elucidate the biological pathways and networks in which differentially expressed genes are involved, providing insights into the functional implications of gene expression changes in specific cellular processes, a thorough examination of Kyoto Encyclopedia of Genes and Genomes (KEGG) enrichment was undertaken to compare European meat pigeons with domestic Yuzhong pigeons, revealing crucial signaling pathways central to breast muscle development. These pathways include carbon metabolism, amino acid biosynthesis, glycolysis/gluconeogenesis, estrogen signaling, PI3K-AKT signaling, endoplasmic reticulum protein processing, oxidative phosphorylation, pentose phosphate pathway, fructose and mannose metabolism, and tight junctions. Notably, the carbon metabolism pathway showed the highest gene involvement, with 22 genes implicated, followed by amino acid biosynthesis with 19 genes associated with breast meat development. Fig 5A provides a concise overview and visualization of these signaling pathways, depicting the number of genes contributing to breast muscle development. Additionally, Fig 5B presents a bubble plot illustrating KEGG signaling pathways and their respective enrichment factors during breast muscle development.

### The GO terms involved in breast muscle development

**Fig 6:**
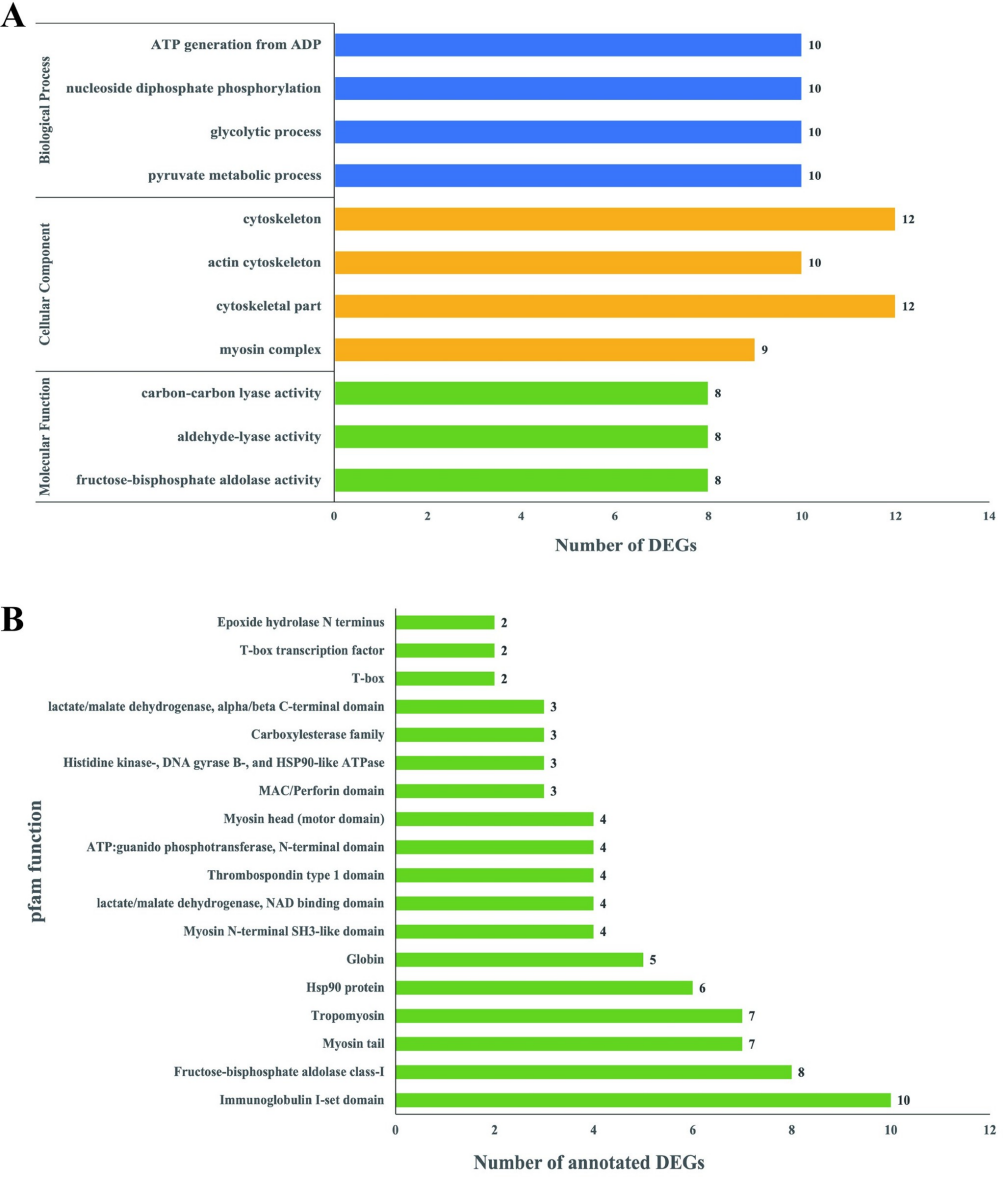
The GO terms and pfam function involved in breast muscle development. (A) GO category, terms, and number of enriched DEGs. (B) The analyzed pfam function and number of annotated DEGs.

To uncover the functional roles of differentially expressed genes and provides insights into the biological significance of gene expression changes observed in the transcriptome data, the molecular function category, significant involvement of fructose-biphosphate aldolase activity, aldehyde-lyase activity, and carbon-carbon lyase activity in breast muscle development is observed, each with 8 annotated DEGs. Regarding the cellular component category, four terms contribute to breast muscle development: myosin complex, cytoskeletal part, actin cytoskeleton, and cytoskeleton, enriched with 9, 12, 10, and 12 DEGs, respectively. Within the biological process category, four terms were also enriched: pyruvate metabolic process, glycolytic process, nucleoside diphosphate phosphorylation, and ATP generation from ADP, each associated with 10 enriched DEGs. Fig 6A offers a concise summary and visualization of the enriched terms, indicating the number of DEGs contributing to breast muscle development.

### The pfam function in breast muscle development

The purpose of Pfam analysis in RNA-seq is to identify conserved protein domains within the identified transcripts. By comparing the sequences against the Pfam database, this analysis helps to elucidate the structural and functional characteristics of the encoded proteins, providing valuable insights into their potential roles in biological processes. In this study, a total of 18 Pfam functions were identified as crucial for breast muscle development. These functions encompassed various domains such as immunoglobulin I-set domain, fructose-bisphosphate aldolase class-I, myosin tail, tropomyosin, Hsp90 protein, globin, myosin N-terminal SH3-like domain, lactate/malate dehydrogenase NAD binding domain, thrombospondin type 1 domain, ATP: guanido phosphotransferase N-terminal domain, myosin head (motor domain), MAC/Perforin domain, histidine kinase-DNA gyrase B- and HSP90-like ATPase, carboxylesterase family, lactate/malate dehydrogenase alpha/beta C-terminal domain, T-box, T-box tran-scription factor, and epoxide hydrolase N terminus. The number of differentially expressed genes (DEGs) annotated for these Pfam functions ranged from 2 to 10. Fig 6B provides a comprehensive summary and visual representation of these Pfam functions, illustrating the number of DEGs contributing to the complex processes involved in breast muscle development.

### Genes involved in breast muscle development

The comparative analysis between European meat pigeons and domestic Yuzhong pigeons uncovered several pivotal genes involved in breast muscle development. This study identified 10 up-regulated and 14 down-regulated differentially expressed genes (DEGs) that actively contribute to this process. Notably, tropomyosin beta chain exhibited the highest up-regulated fold change, followed by fibrinogen gamma chain, with fold change values of 26.181 and 5.614, respectively. Conversely, myosin light chain 3 displayed the most substantial down-regulated fold change, followed by apolipoprotein A-I, with fold change values of 28.136 and 25.791, respectively. Table 4 provide a comprehensive visual representation of the fold change values for these key DEGs during breast muscle development.

**Table 4:** The transcriptional level of annotated DEGs implicated in the breast muscle development.

Genes	Transcription level
Tropomyosin beta chain	+26.181
Fibrinogen gamma chain	+5.614
Myosin regulatory light chain 2	+4.863
Myosin-binding protein C	+4.596
Tropomyosin alpha-4 chain	+4.314
Inter-alpha-trypsin inhibitor heavy chain H3	+4.178
Myotilin	+2.761
Myosin heavy chain	+2.433
Myosin-1	+1.851
Tropomyosin alpha-1 chain	+1.059
Tropomyosin alpha-3 chain	-1.222
Dual specificity protein phosphatase 6	-1.319
Dual specificity protein phosphatase 8	-2.015
Dual specificity protein phosphatase 1	-2.337
Dipeptidyl aminopeptidase-like protein 6	-2.392
Myosin light chain 1	-3.596
Vimentin	-4.094
Actin, alpha skeletal muscle	-4.221
Tropomyosin alpha-1 chain	-4.401
Serine/arginine-rich splicing factor 7	-6.135
Myoglobin	-6.155
Myosin-1B	-6.519
Apolipoprotein A-I	-25.791
Myosin light chain 3	-28.136

Indicator: + represent up-regulated;—represent down-regulated; all transcription level count in log2Foldchange.

### RT-qPCR validation

**Fig 7:**
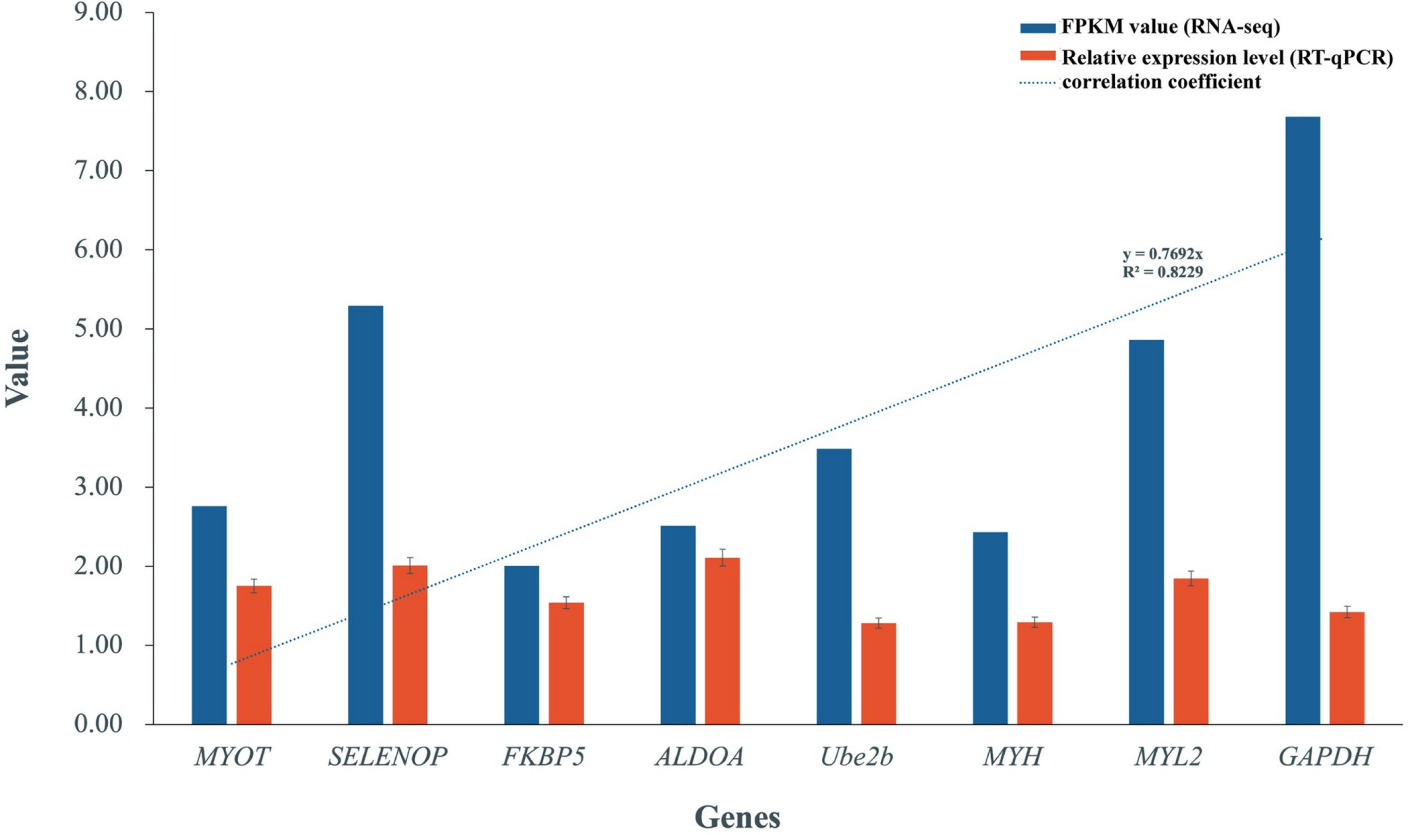
The RT-qPCR validation for the RNA-seq. The error bar denotes the standard error of the mean (SEM).

To validate the accuracy of expression levels obtained from RNA-seq, we employed RT-qPCR to assess the relative mRNA levels of *MYOT*, *SELENOP*, *FKBP5*, *ALDOA*, *Ube2b*, *MYH*, *MYL2*, *and GAPDH*. The transcription levels obtained from RT-qPCR correspond well with the RNA-seq data, demonstrating consistent results between the two methods. These results substantiate the robustness and trust-worthiness of the RNA-seq dataset produced in this study. Fig 7 illustrates the transcription levels and FPKM values for RT-qPCR and RNA-seq, respectively.

## Discussion

Breeders commonly prioritize the cultivation of larger-sized and fuller-breasted pigeons. European meat pigeons, for example, can attain weights of up to 600 g by 28 days of age [30]. Commercially, pigeon meat is typically sourced from squabs aged 28 to 30 days, with body weights ranging from 400 to 700 g [31]. Notably, pigeon carcasses exhibit a higher percentage of breast muscle compared to broiler chicken carcasses [31]. Previous studies have elucidated the genetic mechanisms underlying meat production in various meat pigeon breeds, including European meat pigeons, silver king pigeons, and domestic highflyer pigeons [30]. Additionally, research has investigated developmental and phenotypic disparities as well as differentially expressed genes (DEGs) in the pectoral muscles between European meat pigeons and Shiqi pigeons [32]. Our study found that the total evisceration rate of Yuzhong pigeons was 0.68±0.11%, exceeding that of European meat pigeons, which stood at 0.63±0.55%. Moreover, the breast muscle ratio of Yuzhong pigeons was calculated to be 25.24±4.00%, indicating a higher proportion compared to European meat pigeons, which averaged 22.83±2.63% (unpublished data). Based on these findings, we utilized Iso-seq and RNA-seq methodologies to identify candidate genes influencing pigeon breast muscle development. In this study, we employed Iso-seq and RNA-seq to investigate genes and signaling pathways potentially involved in the development of breast muscles in domestic Yuzhong pigeons. We conducted a comparative analysis of DEGs between European meat pigeons and domestic Yuzhong pigeons in breast muscle development.

Pigeon meat is recognized for its high protein content, medicinal value, and low cholesterol, making it a nutritionally rich food source [4]. Referred to as the ’animal ginseng’ due to its nourishing effects, pigeon meat is gaining increasing attention among consumers in China [4]. In the meat production industry, the performance of pigeon meat serves as a significant indicator of economic value for pigeon species [33]. Therefore, understanding the molecular regulatory mechanisms underlying breast muscle development in pigeons is essential for molecular breeding aimed at enhancing meat production performance. Despite its importance, research on pigeon breast meat development, particularly regarding genes and regulatory mechanisms, remains limited. Consequently, investigating and identifying the genes and signaling pathways involved in regulating breast muscle development is crucial. To address this gap, we employed Iso-seq and RNA-seq techniques to identify these key genes and signaling pathways involved in pigeon breast muscle development.

The third generation PacBio Iso-seq technology represents a powerful sequencing tool for generating full-length transcriptomes, addressing the limitations of short-read transcriptomes produced by Illumina RNA-seq [20]. PacBio Iso-seq has been instrumental in identifying and characterizing novel genes and long non-coding RNAs (lncRNAs), investigating alternative splicing, and elucidating alternative polyadenylation across diverse organisms [34]. Iso-seq and RNA-seq provide invaluable insights into the genetic architecture and expression profiles of pigeons, crucial for enhancing breeding practices. These technologies enable comprehensive characterization of the transcriptome, facilitating the identification of key genes, regulatory elements, and pathways associated with desirable traits such as meat quality, reproductive performance, and disease resistance. By elucidating the molecular mechanisms underlying these traits, Iso-seq and RNA-seq empower breeders to make informed decisions regarding mating strategies, selection criteria, and genetic improvement programs. Additionally, they offer a means to assess the genetic diversity within pigeon populations, aiding in the development of breeding schemes aimed at conserving and optimizing valuable genetic resources. Overall, Iso-seq and RNA-seq play a pivotal role in advancing pigeon breeding practices by providing a deeper understanding of the genetic basis of desirable traits and facilitating more precise and efficient breeding strategies.

Previous research on pigeon meat development has primarily focused on skeletal muscle development, delving into skeletal muscle cell differentiation involving key genes such as *MyoD*, *MyoG*, *MRF4*, and *Myf5* [35]. However, studies aimed at identifying key genes associated specifically with breast muscle development in pigeons have been limited. While genetic insights have been provided, the exploration of their deeper roles has been hindered by a lack of comprehensive sequenced data, underscoring the necessity of acquiring full-length cDNA [36]. In this study, we strategically pooled RNA samples from various tissues of domestic Yuzhong pigeons, including breast muscle, leg muscle, liver, abdominal fat, skin fat, hypothalamus, pituitary, and cecum. This approach yielded a robust collection of unigenes, mitigating Iso-seq errors [37]. Leveraging the Iso-seq technique, this investigation is poised to unravel the intricate facets of domestic Yuzhong pigeon breast muscle development.

This study conducted a comprehensive comparative transcriptomic analysis of breast muscle development in domestic Yuzhong pigeons and European meat pigeons by integrating Iso-seq and RNA-seq techniques. Focusing on the breast muscle, a critical determinant of meat quality and production, we identified 443 DEGs between domestic Yuzhong pigeons and European meat pigeons. Additionally, signaling pathway analysis revealed the breast muscle development network, including pathways such as carbon metabolism pathway, biosynthesis of amino acids, and glycolysis/gluconeogenesis. Molecular function enrichment analysis high-lighted terms such as fructose-biphosphate aldolase activity, while cellular component enrichment included the myosin complex, and the pyruvate metabolic process was enriched in biological process terms. In the pfam analysis, enrichment was observed for fructose-bisphosphate aldolase class-I, myosin tail, and tropomyosin. The transcriptomic analysis reveals key genes and pathways involved in breast muscle development, enhancing our understanding of regulatory mechanisms critical for meat quality and production.

Carbon metabolism is significant for its bioenergetic functions in all cellular processes, including cell signaling, proliferation, and differentiation [38]. Cellular energy metabolism, a component of carbon metabolism, is highly compartmentalized at the subcellular level. Previous studies have revealed notable correlations between metabolic activity and development [39], suggesting that metabolic activities are regulated during development, potentially influencing developmental patterning [40]. Furthermore, research has shown the importance of carbon metabolism in myogenic progression, underscoring its role in normal muscle development [38]. Carbon metabolism is believed to generate ATP [41], which is essential for muscle growth and repair. Moreover, it plays a crucial role in maintaining redox balance; reactive oxygen species (ROS) generated during energy production can damage cellular components [42]. Pathways within carbon metabolism, such as the pentose phosphate pathway, can help neutralize ROS and protect muscle cells from oxidative damage. Carbon metabolism is crucial for its bioenergetic functions in cellular processes, encompassing signaling, proliferation, and differentiation. It’s compartmentalized at the subcellular level, with metabolic activity correlated with development, potentially influencing developmental patterning. Studies highlight its importance in myogenic progression and muscle development, generating ATP essential for growth and repair. Moreover, it maintains redox balance, mitigating ROS-induced damage via pathways like the pentose phosphate pathway, crucial for muscle cell protection. Overall, carbon metabolism is vital for providing energy and regulatory signals essential for muscle tissue growth, maintenance, and function.

Amino acids serve as crucial components of proteins and various bioactive molecules that play regulatory roles in biological processes [43]. Previous studies have indicated that certain amino acids, such as leucine, can enhance muscle protein anabolism [44]. In amino acid metabolism, PGC1α acts as a coactivator of transcription factors, activating the expression of genes involved in fatty acid oxidation, which is essential for regulating muscle fiber formation [45]. Additionally, the intake of amino acids can regulate muscle protein synthesis, and the biosynthesis of amino acids appears to be effective in activating mTORC1 and increasing muscle mass [46]. Amino acids are fundamental constituents of proteins and vital for various regulatory processes in biology. Studies have shown specific amino acids like leucine can enhance muscle protein synthesis. In amino acid metabolism, PGC1α activates genes involved in fatty acid oxidation, crucial for muscle fiber formation. Moreover, amino acid intake regulates muscle protein synthesis, with amino acid biosynthesis activating mTORC1, enhancing muscle mass. Understanding this pathway is pivotal for breast muscle development and production, underscoring amino acids’ significant role in this process.

Tropomyosin beta chain plays a pivotal role in both muscle and meat development. This protein is integral to the regulation of muscle contraction and relaxation, modulating the interaction between actin and myosin within muscle fibers [47]. During muscle devel-opment, tropomyosin beta chain contributes to the formation of muscle structure and the assembly of sarcomeres, the basic contractile units of muscle tissue [48]. Its expression and localization are tightly controlled, influencing muscle fiber type specification [49]. The Tropomyosin beta chain is essential for muscle and meat development, regulating muscle contraction by modulating actin-myosin interaction. It contributes to muscle structure formation, sarcomere assembly, and fiber type specification. Its role extends to im-pacting meat texture, tenderness, and juiciness, making it a significant area of study in muscle biology and meat science.

The combined approach utilizing PacBio Iso-seq and Illumina RNA-seq techniques provides a comprehensive understanding of gene expression patterns and isoform diversity, crucial for deciphering the regulatory mechanisms driving breast muscle development. These methodologies offer detailed insights into transcriptomic alterations, facilitating the identification of pivotal genes and pathways orchestrating muscle growth, differentiation, and functionality. Such analyses enable researchers to unravel the molecular intricacies governing breast muscle development, thereby refining strategies to enhance meat quality and production efficiency. Additionally, this knowledge holds promise for improving domestic pigeon farming practices through the implementation of molecular breeding strategies aimed at augmenting breast muscle yield and overall meat productivity.

## Conclusions

In this study, we employed the PacBio platform to delve into the intricate biology of breast muscle development in domestic Yuzhong pigeons using Iso-seq analysis. Analysis of the Iso-seq dataset yielded a substantial number of subreads, totaling 131,377,075, which subsequently led to the identification of 16,587 non-redundant high-quality full-length transcripts post-correction. Additionally, employing pfam, CPC, PLEK, and CPAT tools, we identified 5575, 4973, 2333, and 4336 lncRNAs, respectively. Through KEGG enrichment analysis, we unveiled crucial signaling pathways associated with breast muscle development, including carbon metabolism, amino acid biosynthesis, glycolysis/gluconeogenesis, estrogen and PI3K-AKT signaling, protein processing in the endoplasmic reticulum, oxidative phosphorylation, pentose phosphate pathway, fructose and mannose metabolism, and tight junctions. These pathways play pivotal roles in regulating breast muscle development, thereby influencing meat quality and production efficiency in domestic poultry farming. These findings offer profound insights into the mechanisms orchestrating breast muscle development in domestic Yuzhong pigeons, setting the stage for further exploration into its dynamics.

## Supporting information

S1 Table DEGs list. (XLSX)

S1 Fig European meat pigeon. (JPG)

S2 Fig Yuzhong pigeon. (JPG)

S3 Fig CCS length. (PNG)

S4 Fig FLNC length. (PNG)

S5 Fig NGS corrected FLNC length. (PNG)

S6 Fig Self-corrected FLNC length. (PNG)
