# Supplementary figures and images for: Unraveling the molecular landscape of breast muscle development in domestic Yuzhong pigeons and European meat pigeon: Insights from Iso-seq and RNA-seq analysis
Source: PLoS One. 2024 Jul 25;19(7):e0305907. doi: 10.1371/journal.pone.0305907 (PMC11271864; doi:10.1371/journal.pone.0305907)

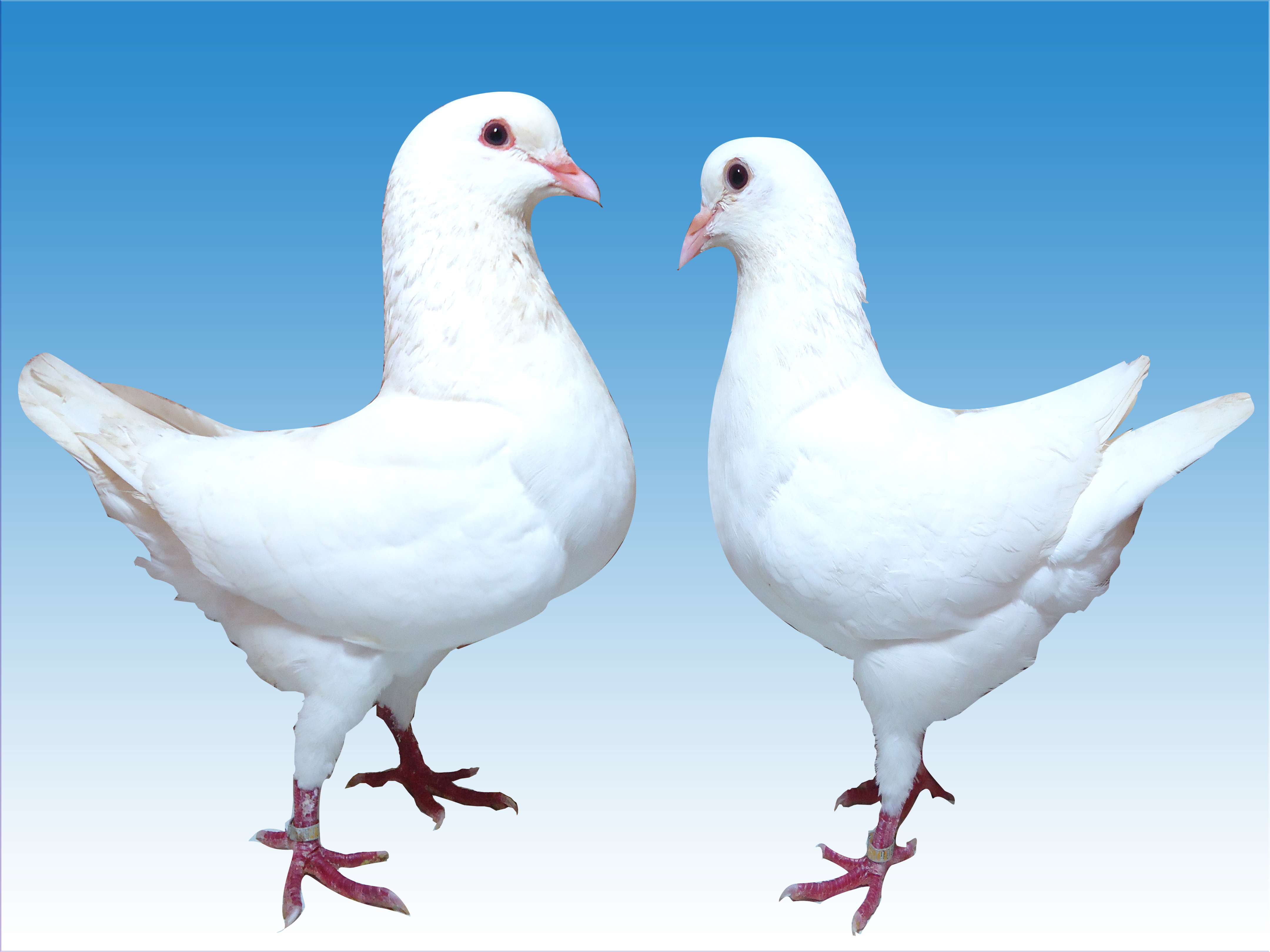

Supplement: S1 Fig — (JPG) [file pone.0305907.s002.jpg]

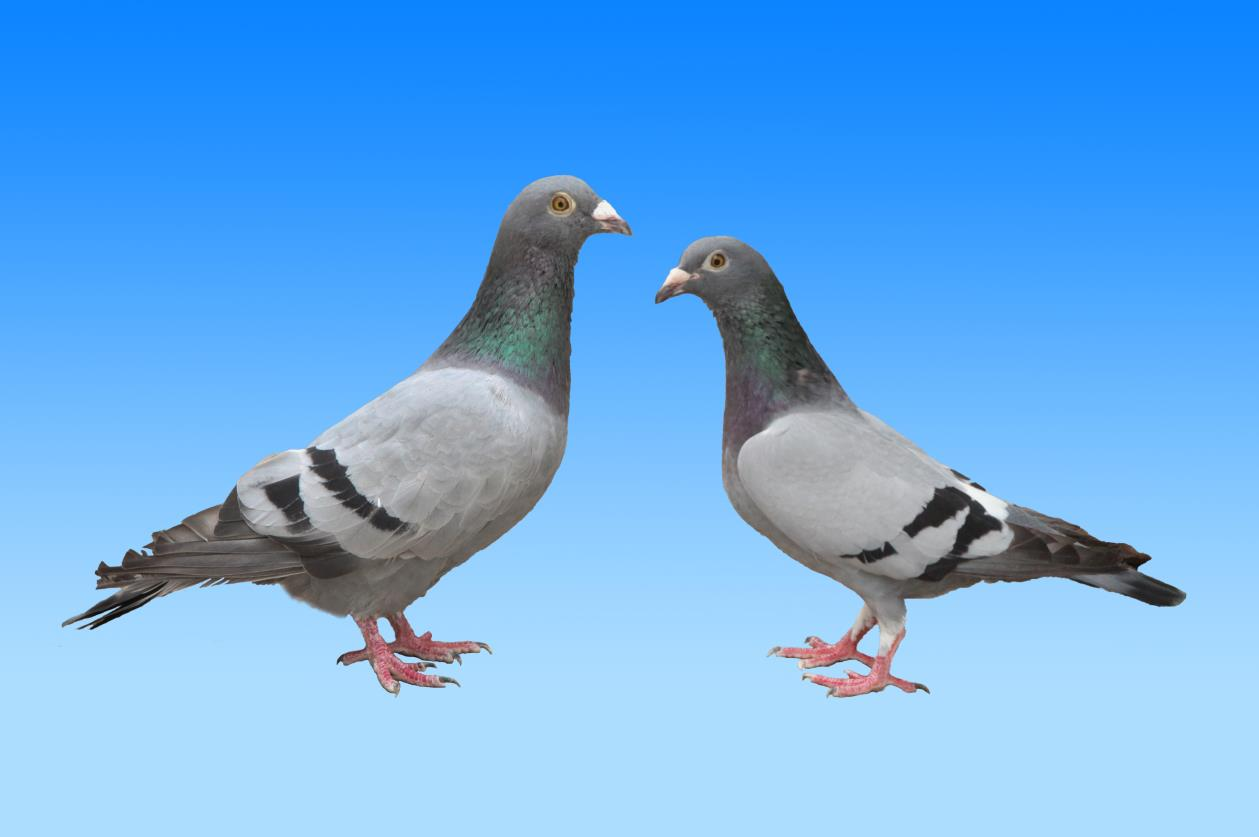

Supplement: S2 Fig — (JPG) [file pone.0305907.s003.jpg]

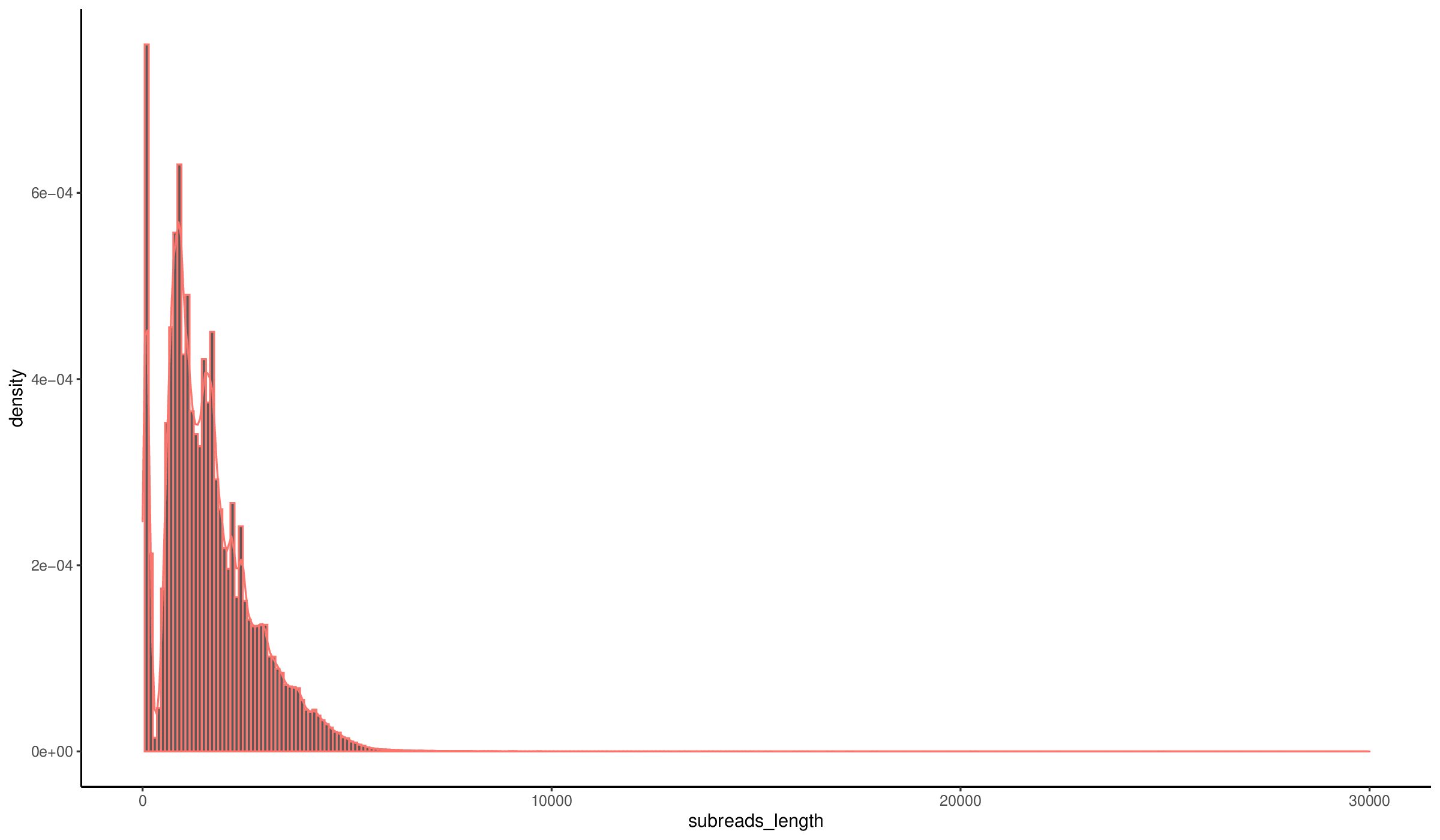

Supplement: S3 Fig — (PNG) [file pone.0305907.s004.png]

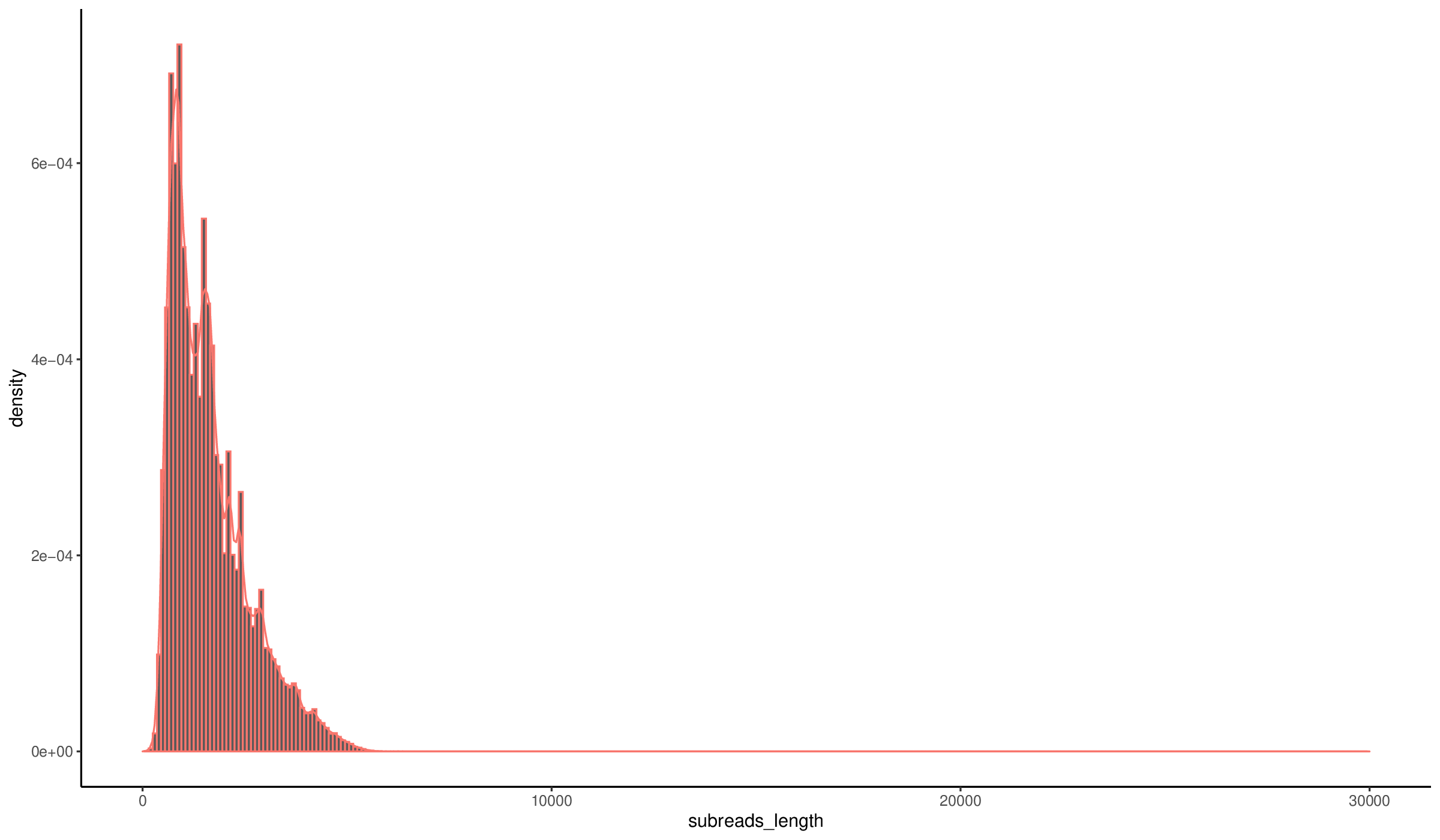

Supplement: S4 Fig — (PNG) [file pone.0305907.s005.png]

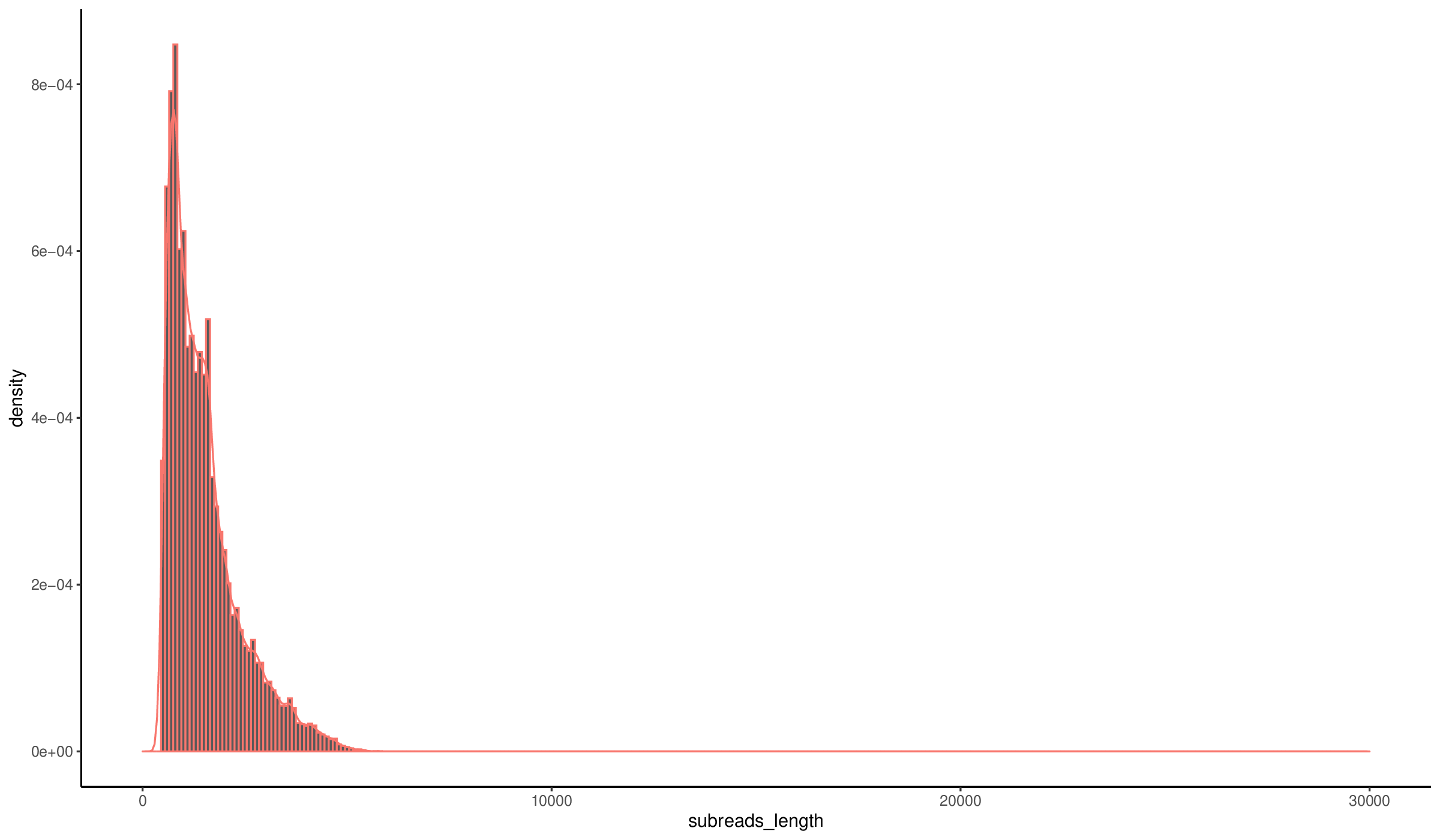

Supplement: S5 Fig — (PNG) [file pone.0305907.s006.png]

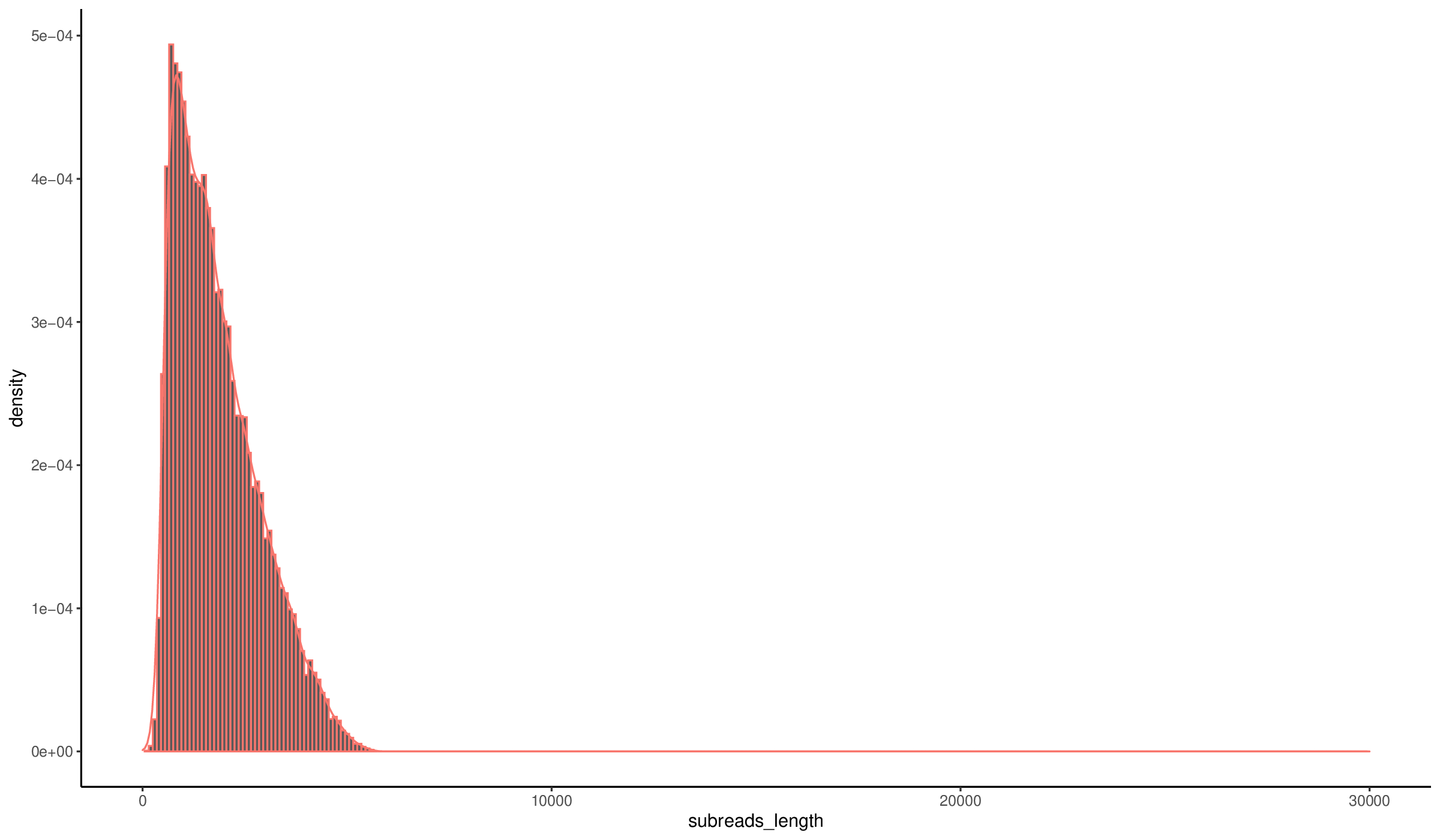

Supplement: S6 Fig — (PNG) [file pone.0305907.s007.png]
